# Contrast-Enhanced Ultrasound for the Characterization of Infantile Hepatic Hemangioma in Premature Neonate

**DOI:** 10.7759/cureus.9580

**Published:** 2020-08-05

**Authors:** Marjan Sekej, Sabina Vadnjal Đonlagić, Damjana Ključevšek

**Affiliations:** 1 Radiology, University Medical Centre Maribor, Maribor, SVN; 2 Pediatric Radiology, University Medical Centre Ljubljana, Ljubljana, SVN

**Keywords:** ceus, contrast-enhanced ultrasound, ihh, infantile hepatic hemangioma

## Abstract

Contrast-enhanced ultrasonography (CEUS) is a relatively new imaging method for use in children. It is recognized as a safe and easily performed problem-solving method.

A premature 10-day-old female infant experienced unusual persistent anemia. The diagnostic workup for the anemia included an abdominal ultrasound examination, which showed pathological formation in the left middle quadrant. MRI was used to further asses the lesion and showed a hypervascular lesion with necrotic areas rising from the left hepatic lobe, mainly showing the pattern that indicates an infantile hepatic hemangioma. Main differential diagnosis, hepatoblastoma, could not be excluded. The crucial examination used to differentiate was CEUS.

## Introduction

Contrast-enhanced ultrasound (CEUS) features of infantile hepatic hemangioma (IHH) are still not widely reported, since the method is relatively new for use in infants [1]. In 2016, the United States Food and Drug Administration approved the ultrasound contrast agent (UCA), Lumason (Bracco Diagnostics Inc., Monroe Township, NJ, USA), for pediatric intravascular applications for evaluating focal liver lesions, but in Europe, where Lumason is marketed as SonoVue (Bracco Imaging S.p.A., Milan, Italy), it is still an off-label use in children [2,3]. The purpose of this article is to present CEUS as a useful, problem-solving method and to inform of its contribution to a better, faster, and safer diagnostic recognition of this relatively rare focal hepatic lesion.

Hepatic tumors in children are relatively rare, accounting for 1% to 4% of all pediatric solid tumors [4]. Infantile hepatoblastoma (HBL) is the most common primary hepatic tumor in children, and IHH is the most common vascular hepatic tumor in children, accounting for 12% of all childhood hepatic tumors, being twice more common in females [4,5].

IHH may undergo spontaneous regression, but it can sometimes be life-threatening due to congestive cardiac failure and/or consumptive thrombocytopenia and coagulopathy (Kasabach-Merritt syndrome). While in asymptomatic cases observation is the recommended course, optimal management strategy for symptomatic patients is still controversial [6]. We report a case of sick neonate with palpable solid abdominal mass and symptomatic anemia.

## Case presentation

A 10-day-old female infant, born prematurely after an uncomplicated 36-week pregnancy and delivery, was admitted to the hospital because of an unusual symptomatic anemia. A clinical exam revealed a palpable solid, painless abdominal mass, measuring up to 3 cm below the lower left rib. She was anemic at birth and had icterus. She received phototherapy for a few days, and was given an erythropoietin and iron supplement. Her hemoglobin remained constantly low, ranging between 80 and 90 g/L.

**Figure 1:**
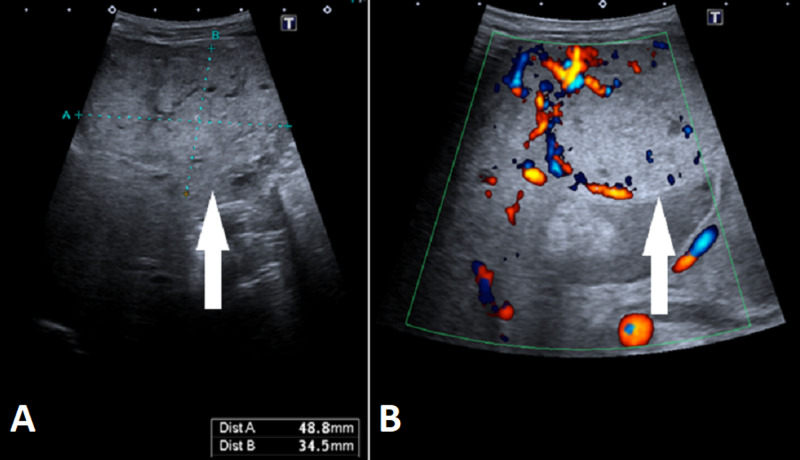
Abdominal ultrasound (US) (A) An abdominal US showing the size of the lesion (white arrow, blue dotted lines – distance A and B). (B) An abdominal Doppler US showing a hypervascular lesion (white arrow) with predominant peripheral vascularization.

**Figure 2:**
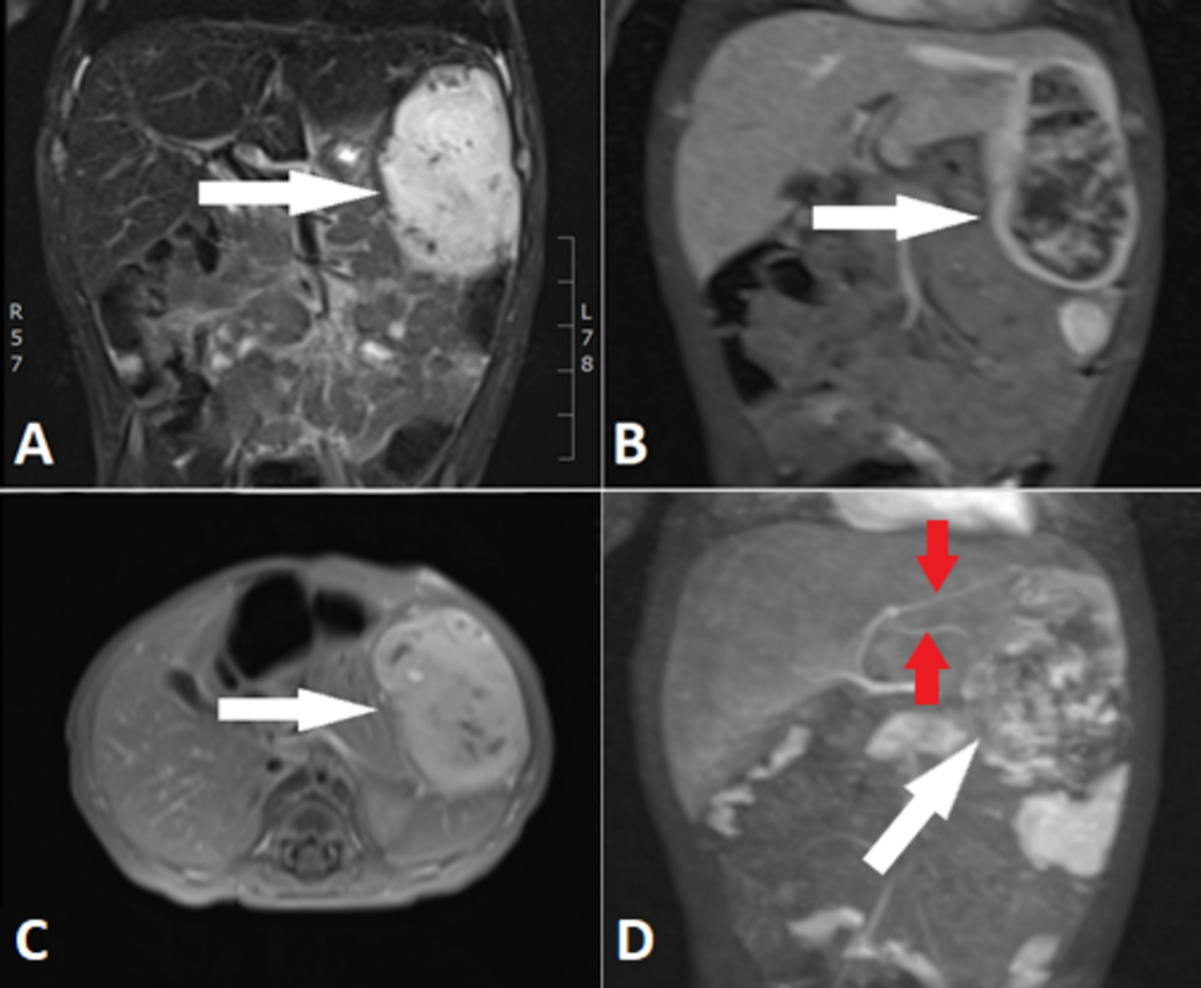
Abdominal MRI An abdominal MRI showing a focal hepatic lesion (white arrow) in different phases and sequences. (A) A coronal T2 fat sat sequence showing a heterogeneous hyperintensive focal lesion rising from the left hepatic lobe. (B) Coronal T1 fat sat vibe contrast medium showing a peripherally enhancing lesion with hypointense central part in the arterial phase. (C) Oblique T1 fat sat late contrast-enhanced venous phase showing a lesion completely filled with contrast medium with same hypointense necrotic areas. (D) Maximum intensity projection (MIP) reconstruction of arterial phase showing two feeding arteries (red arrows).

Ultrasound (US) of the heart at birth showed persistently patent arterial duct and oval foramen without hemodynamic significance. An abdominal US with Doppler (Figure 1 ) and MRI (Figure 2) of the abdomen showed a hypervascular lesion bulging from the left hepatic lobe, with rim enhancement and partial necrosis.

**Figure 3:**
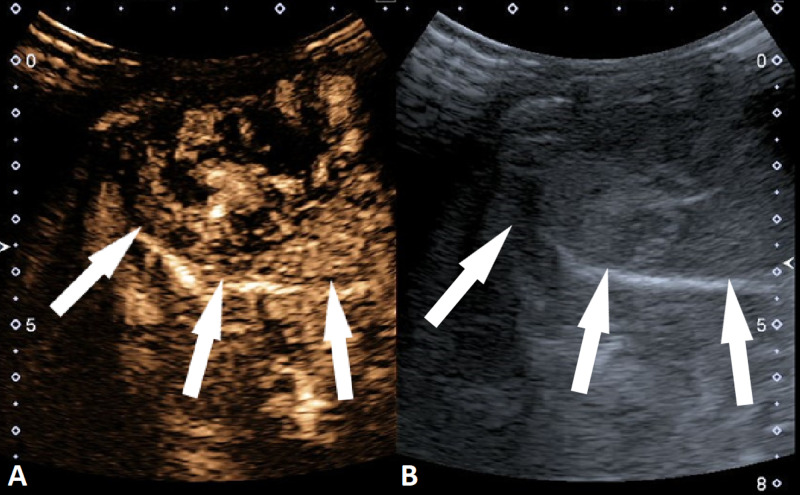
Abdominal contrast-enhanced ultrasound (CEUS) (A) An abdominal CEUS showing peripheral nodular enhancement of the lesion (white arrows) eight seconds after IV contrast administration; (B) parallel to the CEUS, a gray-scale US of the lesion (white arrows).

**Figure 4:**
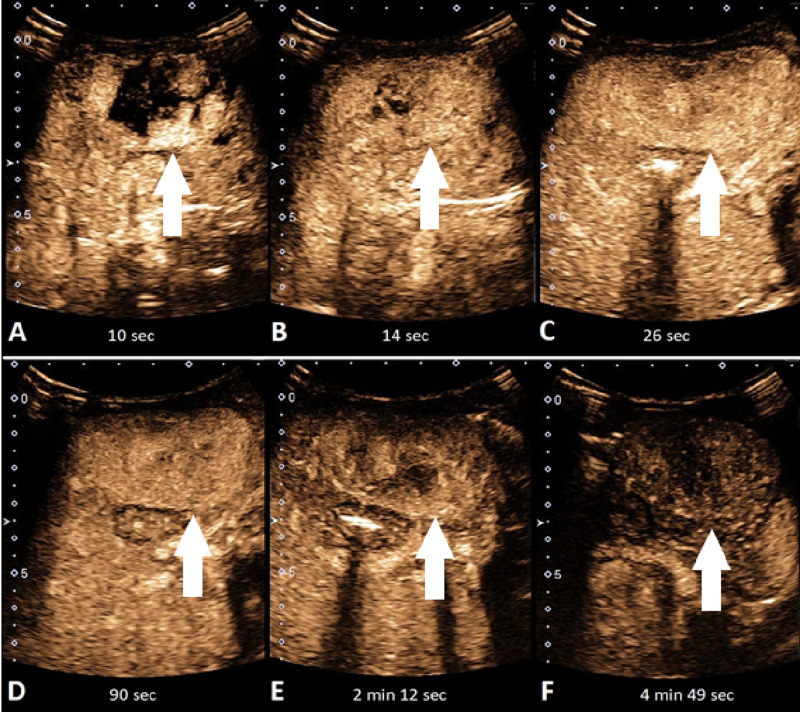
Abdominal contrast-enhanced ultrasound (CEUS) phases A liver CEUS at different times after IV contrast administration: (A) peripheral enhancement of the lesion after 10 seconds, (B, C) fast and complete filling of the lesion with contrast medium in the late arterial phase (14 and 26 seconds), (D-F) completely filled lesion with contrast medium remaining slightly hyperenhanced compared to normal liver tissue  in the venous and portal phase (90 seconds, 2 minutes and 12 seconds, 4 minutes and 49 seconds).

The blood supply to this focal hepatic lesion came from two feeding arteries rising from the left hepatic artery. There were still some issues in differential diagnosis between HBL and IHH due to slightly elevated serum alpha-fetoprotein (AFP). Therefore, an intravenous CEUS of the focal hepatic lesion was performed. The UCA SonoVue with a dose of 0.15 mL (0.05 mL/kg) was used. On CEUS, a distinctive enhancement pattern of the rapid-filling hepatic hemagioma is shown; peripheral nodular enhancement starting immediately in arterial phase (eight to ten seconds after IV administration of UCA) with centripetal filling completed in eight seconds (only small necrotic areas remained non-enhanced) (Figure 3). The lesion remained slightly hyperenhanced in the venous and portal phase (Figure 4). There were no signs of wash out. There were no signs of lymphadenopathy or metastasis.

Serum AFP was slightly elevated at the beginning and decreased over time. Serum concentrations of tumor markers neuron-specific enolase and human chorionic gonadotropin were normal. Concentrations of vanillylmandelic acid and homovanillic acid in the urine were normal. Kasabach-Merritt syndrome was also ruled out since the platelet count was normal. The brain US showed bilateral lenticulostriate vasculopathy.

Treatment with propranolol at the age of four weeks was started, after which an abdominal US showed a minor regression in the lesion size, but hemoglobin remained low even two months later.

## Discussion

The diagnostic approach to infant liver focal lesions is challenging and requires a complete workup because of the symptoms and concerns about malignancy. Diagnostic imaging modalities may facilitate a differentiation between benign and malignant liver tumors, or may even be a crucial method for establishing the right diagnosis on the basis of the enhancement pattern [7].

The first imaging modality commonly used for focal hepatic lesions is the abdominal US with a color Doppler to determine the origin of the lesion and its gross structure and vascularization [8,9]. The next step in imaging evaluation is contrast-enhanced MRI, which can give more detailed information on the structure of the lesion, better anatomical localization, staging, a more accurate analysis of the relationship with the surrounding parenchyma than US, the metabolic pattern of its tissue, and involvement of the local lymph nodes. Contrast-enhanced CT scan can also give valuable information about the lesion, but carries a big dose burden for an infant and should be avoided [3]. Finally, CEUS is a relatively new imaging method for infants, which could provide diagnostic performance similar to other imaging modalities in focal liver lesions, and has multiple advantages over CT and MRI: it is a radiation-free and sedation-free method, it is readily available and easily repeatable, and it is not as sensitive to motion artifacts as CT and MRI [2,9]. The temporal resolution with CEUS provides real-time insight into the internal vascularity of a lesion. As a problem-solving method with a high safety profile, it is recommended to be performed immediately after classical US, before MRI or CT. In most cases, CEUS is diagnostic, and therefore there is no need for an MRI/CT or fine needle puncture.

Second-generation UCAs are on the market now and consist of microbubbles composed by inert gas surrounded by a phospholipid or albumin coat. They are a blood pool agent, which means that microbubbles remain in the vessels and do not diffuse in extravascular space. The safety profile of UCA is high with very low incidence of side effects [2,9]. The frequency of adverse events associated with the intravenous use of UCA is lower than the one associated with the use of iodinated contrast material at CT and similar to the one associated with the use of gadolinium-based contrast agents [2,10]. However, the long-term effects related to gadolinium-based contrast material deposition in brain tissue are unknown, particularly in small immature babies.

The characterization of a lesion during different phases of enhancement improves diagnostic accuracy and treatment. Recently, El-Ali et al. described the CEUS pattern of five cases of infantile and five cases of congenital hemangioma [1]. One should be aware that the pattern for the enhancement of IHH differs from the one in adult’s hemangioma. In adults, the typical hepatic hemangioma shows peripheral nodular enhancement in the arterial phase with complete (sometimes incomplete) centripetal filling in the portal venous and late phases. In IHH, the filling phase of hemangioma is rapid and is usually completed at the end of the arterial phase or at the beginning of the venous phase. It usually remains isoenhanced or slightly hyperenhanced with surrounding liver parenchyma [1,3].

Abdominal mass and anemia are common indicators of both HBL and IHH, with AFP being the most important clinical marker for HBL, and remains the key clinical marker of malignant change, response to treatment, and relapse. However, there are some variants of both HBL and hepatocellular carcinoma that have low or normal AFP levels [5]. AFP levels are rarely elevated above the normal reference range for age in IHH patients [11].

In our case, there were certain doubts about differential diagnosis after MRI, and due to the slightly elevated serum AFP and persisting anemia, CEUS was performed. Since the results showed the typical pattern of rapid filling hemangioma, we could confirm the MRI findings and conclude with certainty that the diagnosis is IHH, rather than HBL.

## Conclusions

Diagnosis of a neonate abdominal mass is challenging due to the urge to begin with therapy, and possible complications that may suddenly occur if left untreated. CEUS should be taken into account as an equally reliable method as MRI for diagnostic differentiation of focal hepatic lesions in infants, sparing time, money, and complications tied to gadolinium contrast adverse effects and general anesthesia, which is required for MRI. IHH should be considered in the differential diagnosis of infants with an abdominal mass in the newborn period as it could be associated with significant morbidity and mortality, requiring aggressive treatment and meticulous supportive care.
